# Maternal and placental galectins: key players in the feto-maternal symbiotic tango

**DOI:** 10.1007/s00281-025-01061-w

**Published:** 2025-08-21

**Authors:** Orsolya Oravecz, Yiran Xie, Andrea Balogh, Máté Posta, Charlotte Harms, Emese Farkas, Sophia Borowski, Júlia Szekeres-Barthó, Nándor Gábor Than, Sandra M. Blois

**Affiliations:** 1 Systems Biology of Reproduction Research Group, Institute of Molecular Life Sciences, HUN-REN Research Centre for Natural Sciences, H-1117 Budapest, Hungary; 2 Doctoral School of Biology, ELTE Eötvös Loránd University, Budapest, H-1117 Hungary; 3 Department of Obstetrics and Fetal Medicine, University Medical Center Hamburg-Eppendorf, Hamburg, 20251 Germany; 4 Doctoral College, Károly Rácz Conservative Medicine Division, Semmelweis University, Budapest, H-1085 Hungary; 5 Glyco-HAM, a cooperation of Universität Hamburg, Technology Platform Mass Spectrometry and University Medical Center Hamburg-Eppendorf, Hamburg, 20251 Germany; 6 Charité—Universitätsmedizin Berlin, corporate member of Freie Universität Berlin, Humboldt-Universität zu Berlin, and Berlin Institute of Health (BIH) and Institute of Biochemistry, Berlin, Germany and Deutsches Zentrum für Herz-Kreislauf-Forschung (DZHK), partner site Berlin, Berlin, 10117 Germany; 7 Department of Medical Biology and Central Electron Microscope Laboratory, Medical School, University of Pécs, Pécs, H-7624 Hungary; 8 National Laboratory on Human Reproduction, University of Pécs, Pécs, H-7624 Hungary; 9 HUN-REN–PTE Human Reproduction Research Group Hungary, University of Pécs, Pécs, H-7624 Hungary; 10 Department of Obstetrics and Gynecology, Medical School, Semmelweis University, Budapest, H-1088 Hungary; 11 Maternity Private Clinic of Obstetrics and Gynecology, Budapest, H-1126 Hungary

**Keywords:** Decidua, Glycan-binding protein, Obstetrical syndromes, Placenta, Pregnancy galectinology

## Abstract

Galectins, a family of β-galactoside-binding proteins, are critical in regulating feto-maternal interactions during pregnancy. Their evolutionary trajectory is reflected in their expression patterns and diverse functions in embryo implantation, trophoblast invasion, and maternal immune and vascular adaptation, contributing to healthy placentation and uncomplicated pregnancy. Galectin-1 (gal-1), one of the most ancient galectins, plays a pivotal role in feto-maternal immune regulation, acting predominantly from the maternal side to promote immune tolerance, a function integrated early in placental mammalian evolution. In contrast, anthropoid primates introduced a unique set of fetal (placental) galectins (gal-13, gal-14, and gal-16) through birth-and-death evolution, with these genes localized on human chromosome 19. Notably, these primate species have evolved varying degrees of deep placentation, with humans exhibiting the deepest, which facilitates enhanced nutrient delivery to the fetus, particularly for brain development. Placental galectins have been implicated in the evolution of immune tolerance mechanisms that support deep placentation. During pregnancy, reduced expression of maternal galectins (e.g., gal-1) and placental galectins (e.g., gal-13) has been associated with severe obstetric complications, signaling disruptions in feto-maternal tolerance. This review provides a comprehensive overview of gal-1, gal-13, gal-14, and gal-16, highlighting their shared and unique roles in maternal and placental immune regulation and placental development. Additionally, the review explores the potential of maternal versus placental galectins as biomarkers and therapeutic targets to improve diagnostic and treatment strategies for adverse pregnancy outcomes.

## Introduction

Glycan-binding proteins (GBPs) contribute to immunity by interpreting glycocode-encoded information, which in turn triggers cellular signaling, facilitates cell‒cell adhesion, and mediates protein‒protein interactions. Among these, galectins are a distinct subfamily of lectins with specialized functions at the feto-maternal interface. Defined by their carbohydrate-binding properties, galectins possess carbohydrate-recognition domains (CRDs), conserved sequences of 120–140 amino acids, that specifically bind β-galactoside-containing glycoconjugates [1]. First identified as “S-type lectins” by Drickamer, with the “S” designation referring to their sulfhydryl dependency (due to cysteine residues) and solubility properties [2]. To date, 21 galectins have been identified in mammals, with 15 of these found in humans [3]. These proteins are ancient, with homologous galectins identified in early Bilateria, underscoring their evolutionary importance [4, 5]. Galectins are classified into three structural categories: (1) Proto-type galectins (gal-1, -2, -5, -7, -10, -11, -13, -14, -15, -16, -17, -19, and -20), which contain a single CRD capable of dimerizing; (2) Tandem-repeat-type galectins (gal-4, -6, -8, -9, -9b, -9c, and -12), which feature two homologous CRDs connected by a short linker, enabling multivalent carbohydrate binding; and (3) Chimera-type galectin (gal-3), which contains both a C-terminal CRD and an N-terminal non-lectin domain, critical for multimerization and functional regulation [6]. Despite variations in amino acid sequences, galectins share a conserved CRD topology known as a “jelly roll” structure, comprising antiparallel β-sheets, and a set of eight highly conserved residues that facilitate glycan binding through hydrogen bonding, electrostatic, and van der Waals interactions [7, 8]. While most galectins preferentially bind β-galactosides, a few, like gal-10 (Charcot-Leyden crystal protein), show a higher affinity for β-mannosides [9, 10]. Additionally, certain galectins (e.g., gal-1, -2, -3, and -13) also bind glycans with ABO blood group antigens, contributing to their hemagglutinating activity [11].

Galectins are synthesized on free ribosomes in the cytoplasm and are exported to the extracellular environment through unconventional secretion mechanisms, such as direct transport or incorporation into extracellular vesicles (EVs) [12, 13]. Intracellularly, galectins regulate vital cellular processes, including growth, survival, transcriptional regulation, and mRNA splicing [14–17]. Upon secretion, they engage in protein–glycan interactions with the cell surface, extracellular matrix, and pathogens, modulating key processes such as cell adhesion, migration, invasion, apoptosis, and microbial killing—critical for both innate and adaptive immune responses during healthy pregnancy [18–20]. These interactions make galectins unique regulators of host immune responses, inflammation, and immune tolerance [21].

In this review, we focus on prototype gal-1 and placental galectins (gal-13, gal-14, and gal-16), emphasizing their complementary roles at the feto-maternal interface. We discuss their essential functions in regulating feto-maternal tolerance and placenta development as well as their implications for pregnancy outcomes.

## Galectin-1 and placental galectins’ expression and secretion at the feto-maternal interface

**Fig. 1:**
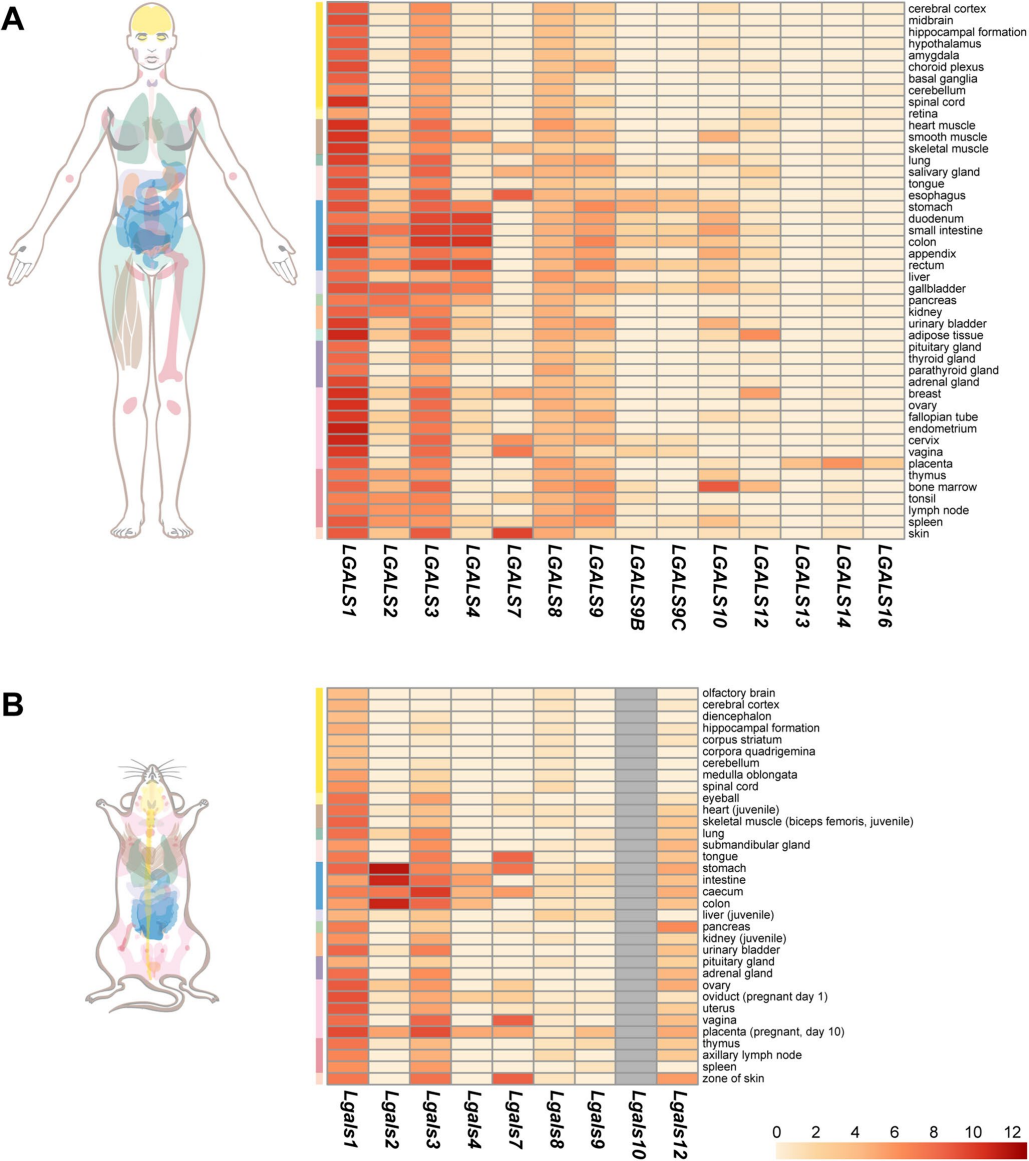
Expression of galectins in humans and mice. Transcript expression levels of members of the *LGALS*/*Lgals* gene family in humans (**A**) and mice (**B**) in various tissues are depicted on heatmaps. Data were downloaded from the Human Protein Atlas (consensus data of the HPA and GTEx projects [29]) and Expression Atlas (FANTOM5 project data) and were visualized after log-transformation [humans: log_2_(nTPM + 1), mice: log_2_(TPM + 1)]. *LGALS1*/*Lgals1* and *LGALS3*/*Lgals3* have the widest and most abundant expression, while *LGALS13*, *LGALS14*, and *LGALS16* expression is restricted to the placenta

Galectins exhibit distinct, yet overlapping tissue expression patterns across mammals, with gal-1 and gal-3 being the most widely distributed, particularly in the endometrium, decidua, and placenta [22–24] (Fig. 1). Gal-1 expression is most prominent in the endometrium and decidua, where it peaks during the late secretory phase of the menstrual cycle and increases during decidualization [24]. This pattern also applies to extravillous trophoblasts (EVTs) within the placenta. Elevated *LGALS1* (encoding gal-1) expression across decidual stromal cell trajectories is further confirmed by recent single-cell RNA sequencing (scRNA-seq) data from healthy pregnancies [25]. Notably, gal-1 expression aligns with the implantation window in the early stages of pregnancy and is controlled by sex steroids [26]. In a fetal loss mouse model, decreased gal-1 levels coincided with reductions in progesterone (P4) and progesterone-induced blocking factor (PIBF), while recombinant gal-1 restored these levels, suggesting a synergistic role for gal-1 and P4 in protecting pregnancy [27]. Additionally, gal-1 is selectively regulated in uterine natural killer (uNK) cells, which are crucial for trophoblast invasion and vessel remodeling during placentation, distinguishing them from peripheral NK cell subtypes [28]. While gal-1 is expressed in all layers of the fetal membranes, villous cytotrophoblasts (CTBs) and EVTs, stromal cells, and the villous endothelium, its levels are lower than in the decidua [10, 22]. These findings highlight the maternal regulation of gal-1 expression during gestation, primarily controlled by sex steroids in the endometrium and decidua. Consequently, maternal-driven gal-1 expression plays a critical role in placentation, immune tolerance, and pregnancy maintenance.

Among placental galectins, the first was identified by Hans Bohn et al., who originally isolated placental protein 13 (PP13) from human placental tissue [30, 31]. In the late ’90s, PP13 cDNA was cloned and then PP13 was identified as gal-13 in a study that confirmed its placenta-specific expression pattern in the human body [32]. Subsequently, a whole subfamily of galectins that includes gal-13, gal-14 and gal-16 was discovered in a chromosome 19 (Chr19) cluster, the largest galectin gene cluster, in which genes have a similar expression pattern at the feto-maternal interface, predominantly expressed in the villous placenta, particularly in the syncytiotrophoblast (STB) [10, 33]. Recent scRNA-seq studies have confirmed the placental-enriched expression of *LGALS13*,* LGALS14*, and *LGALS16* (shortened gene names of placental galectins), with differential expression across various regions of the feto-maternal interface [34–37]. Notably, placental galectin expression is conserved in placentas of anthropoid primates, suggesting an evolutionary significance [10, 38]. Indeed, key transcription factors (TFs) that regulate placental galectin expression during trophoblast differentiation bind to long interspersed nuclear element (LINE) and ALU (Alu elements, which are a type of short interspersed nuclear element (SINE) found in primate genomes) noncoding repeat elements in these genes’ upstream regions, a process that emerged from the insertion of these repeats into the 5’ flanking region of an ancient anthropoid galectin gene [39].

Maternal and placental galectins can enter the circulation, likely through tissue leakage, though their precise role in the bloodstream remains unclear. Soluble gal-1 and gal-13 levels increase steadily from the first to the third trimester, and decline (gal-1) or disappear completely (gal-13) post-delivery, supporting that their increase in maternal blood originates from the placenta [11, 23, 38, 40, 41]. However, recently, using a maternal and placental gal-1 deficient mouse pregnancy model, we demonstrated that placental-derived gal-1 is proportionally limited compared to maternal-derived gal-1 during gestation [22], highlighting that gal-1 in circulation is predominantly derived from the maternal response (e.g., decidua, immune cells and endothelial cells) to pregnancy, rather than leaking from the placenta. Additionally, ABO blood group status can influence the bioavailability of galectin levels in circulation, as has been found for gal-13 [11]. The levels of gal-14 and gal-16 in maternal blood are still undetermined due to the lack of reliable and sensitive assays, e.g., in case of ELISA method, highly specific antibody pairs are required. Furthermore, unlike gal-13 and gal-14, gal-16 has only been detected in placenta at the RNA but not protein level, probably partly because of its very weak expression compared to other placental galectins [10]. Further research in this area should clarify the role of galectins in maternal circulation. In particular, identifying the galectin interactome in the bloodstream may reveal key pathways through which glycan-mediated interactions contribute to immune tolerance during pregnancy.

## The sweet network at the feto-maternal interface and its role in healthy pregnancy

### Galectin-1 and placental galectins are determinants of feto-maternal immune tolerance

**Fig. 2:**
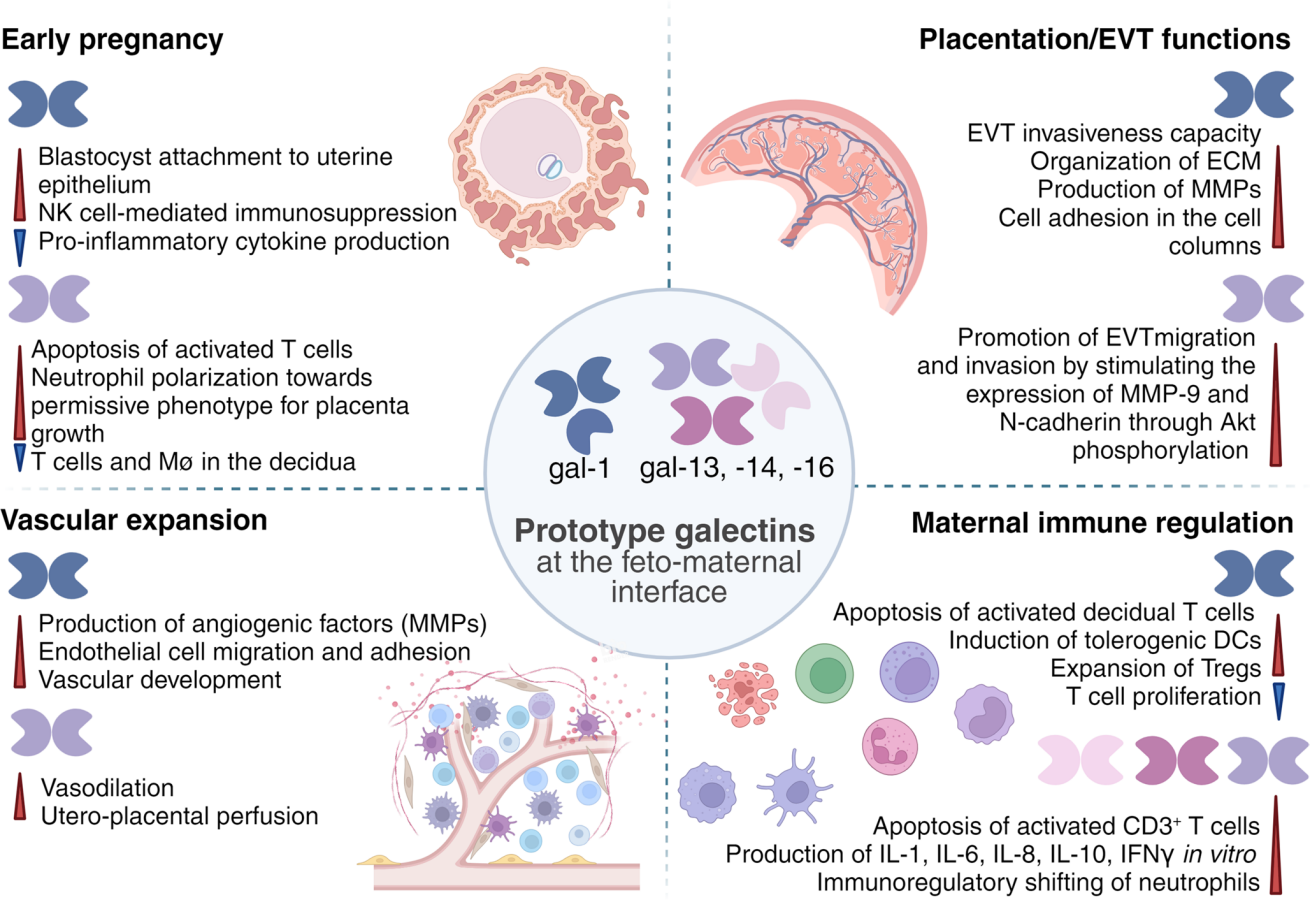
Physiological roles of gal-1, gal-13, gal-14, and gal-16 at the feto-maternal interface. The figure represents multiple regulatory roles of gal-1, gal-13, gal-14, and gal-16 in pregnancy: (1) early pregnancy, including implantation and placental growth; (2) placentation and EVT invasion; (3) vascular expansion, including angiogenesis and vasodilation; and (4) maternal immune regulation, including various feto-maternal immune tolerance mechanisms. DC, dendritic cell; ECM, extracellular matrix; EVT, extravillous trophoblast; gal, galectin; IL, interleukin; MMP, matrix metalloproteinase; Mө, macrophage; NK, natural killer; PBMC, peripheral blood mononuclear cell; Treg, regulatory T cell

**Fig. 3:**
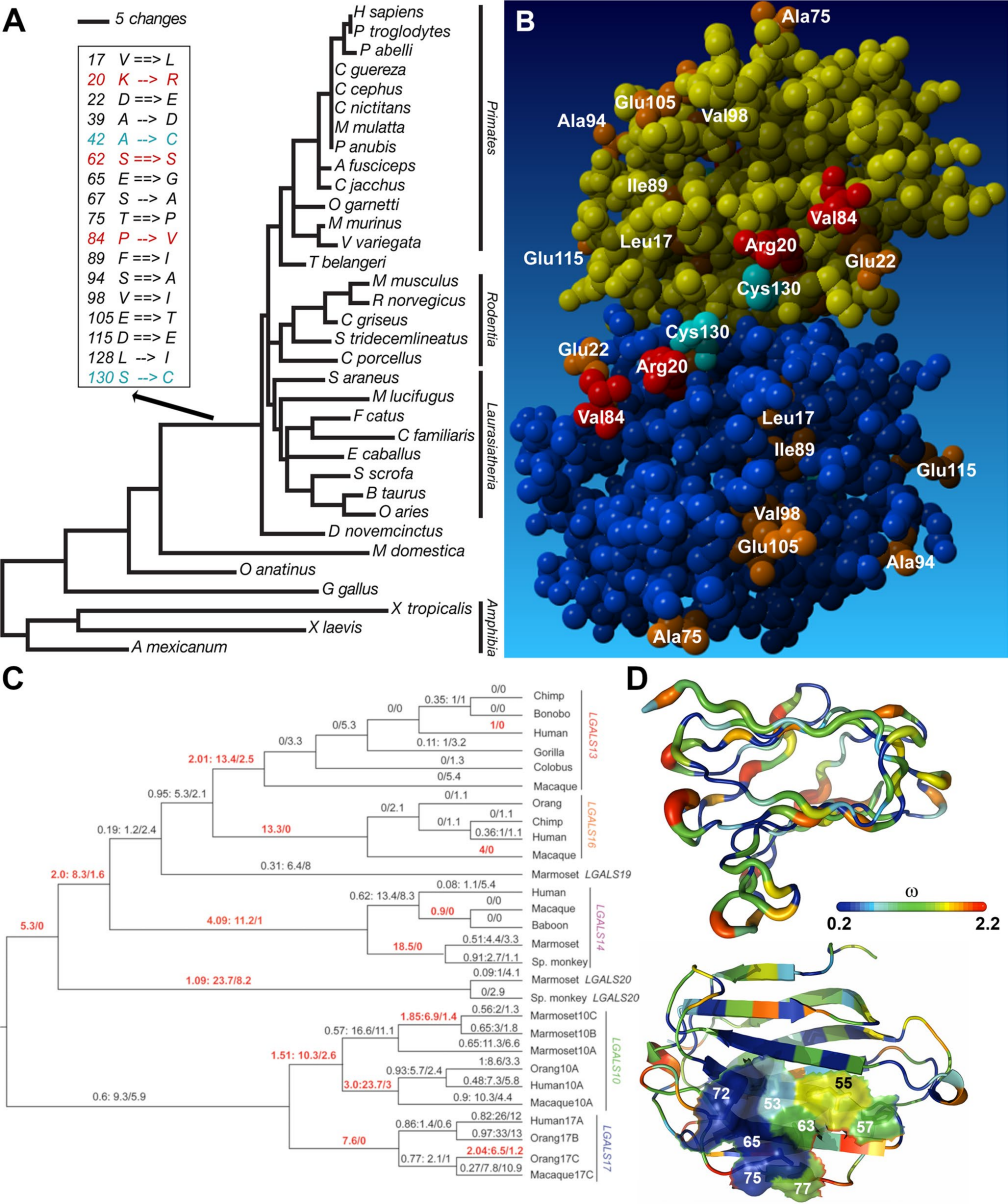
Structure and carbohydrate-binding of gal-1 and placental galectins. (**A**) Phylogenetic tree of gal-1 as published in [26]. Amino acid replacements in the placental stem lineage are presented, and positively selected residues are highlighted in red. Derived cysteines (cyan blue) can disable carbohydrate binding and dimerization upon oxidation due to the formation of disulfide bridges. (**B**) Amino acids replaced on the placental stem lineage are shown in orange on the X-ray crystallographic (1GZW) space-fill model of human dimeric gal-1. Positively selected residues are red, and Cys-130 is cyan blue. Two positively selected residues (Arg-20 and Val-84), along with Cys-130 and Glu-22, form a subdomain next to the dimerization interface. (**C**) In the gene tree of Chr19 cluster galectins published in [10], the numbers above the branches represent ω, N*dN (nonsynonymous substitutions), and S*dS (synonymous substitutions), respectively, shown in red on branches with evidence for positive selection. (**D**, upper) The width of the ribbon representing the gal-16 molecular backbone varies in proportion to site-specific ω values for all cluster galectins. ω, indicated by the color spectrum depicted on the bar, is the highest in loop regions and smallest along-strands. (**D**, lower) The same colour coding shows that four residues in the carbohydrate recognition domain of Chr19 cluster galectins are under strong purifying selection, while others on the opposite side show more variability. Figures adapted from [10, 26], © National Academy of Sciences of the U.S.A

Galectins regulate immune cell functions and balance since the formation of galectin–glycoprotein lattices on immune cell surfaces is important for cell signaling and receptor turnover, thus influencing the decision between cellular survival, proliferation, differentiation, and regulation of immune tolerance or responsiveness [42] (Fig. 2). Fluctuations in redox status can modulate galectins’ conformation, affecting their biological activities. For instance, gal-1 and placental galectins are regulated by redox changes due to cysteine residues in their structure, which have been conserved through placental mammalian evolution [26, 43] (Fig. 3).

Within the maternal immune system, gal-1 is synthesized and secreted by a wide range of cells, including NK cells [44], macrophages [45], and tolerogenic dendritic cells (DCs) [46]. The multifaceted roles of gal-1 across immune cell subsets converge to orchestrate a comprehensive framework of feto-maternal tolerance. In the first half of pregnancy, the predominant population of maternal leukocytes at the feto-maternal interface is uNK cells, while their number decreases during the second half of pregnancy [47]. By expressing growth factors, angiogenic factors, and cytokines, uNK cells contribute to the remodeling of the decidua and spiral arteries, thus increasing the availability of maternal blood at the implantation site and promoting EVT invasion [48, 49]. Gal-1 is expressed by all NK cell types, but uNK cells exhibit increased gal-1 expression compared with circulating NK cell subpopulations [44]. uNK cells contain granules encasing perforin and other cytotoxic proteins. Inflammation may change uNK cell quiescence, leading to abnormal activation and release of granule contents [50, 51]. Clemente and coworkers showed that gal-1 is present in cytotoxic granules of human NK cells, and in vivo cytotoxic assays in *Lgals1*-null mice revealed a novel role for this lectin as an integral component of the lytic machinery of cytotoxic T lymphocytes [52]. We previously showed that maternal-derived *Lgals1*-null mice are associated with aberrant uNK activation characterized by elevated release of cytotoxic perforin granules during the preplacental period. Moreover, maternal *Lgals1* knockout dams displayed fewer vessel-associated uNK cells and exacerbated placental inflammation [22], highlighting gal-1 as a gatekeeper of uNK quiescence. Intriguingly, while gal-1 localizes within the granules of uNK cells, its direct involvement in degranulation remains poorly understood. Future studies leveraging single-cell transcriptomics or conditional knockout models could unravel the role of gal-1 in NK cells’ granules, offering therapeutic insights into pregnancy complications linked to uNK dysregulation.

Macrophages of the innate immune system infiltrate the decidua and the myometrium to establish and maintain feto-maternal tolerance. They are scattered throughout the decidua during early pregnancy, where they become the largest population of immune cells by the end of pregnancy [53, 54]. While classically activated M1 macrophages show antimicrobial and inflammatory effects, alternatively activated M2 macrophages induce tolerance and the resolution of inflammation. During the peri-implantation period, decidual macrophages are skewed toward M1 macrophages, and they transition to a profile of mixed M1 and M2 macrophages when EVTs invade the uterine stroma; then, this mixed population shifts toward a predominantly M2 phenotype to prevent immune-mediated fetal loss and ultimately plays a role in parturition. In M1 macrophages, Toll-like receptors (TLRs) with several N-linked glycosylation sites have been suggested to play important roles in the immune regulation of the female reproductive tract [55, 56]. Amith et al. demonstrated that the desialylation of TLR4, specifically the removal of α2,3-linked sialic acid residues, is a prerequisite for ligand-induced receptor activation. Gal-1 interferes with this critical post-translational modification by preserving α2,3-sialylation, thereby attenuating TLR4 signaling. This inhibition promotes macrophage polarization toward an anti-inflammatory M2 phenotype, a transition pivotal for the resolution of inflammation and the maintenance of fetal immune tolerance [57]. Using gal-1-deficient pregnant mice, we demonstrated increased susceptibility to complete implantation failure following stress challenges during gestation. This phenotype was reversed by lipopolysaccharide (LPS) neutralization, underscoring the pivotal role of gal-1 in regulating LPS/TLR4-mediated immune responses during early pregnancy [58]. In vivo analysis of inflammation sites showed that gal-1 is also crucial for regulating the major histocompatibility complex (MHC)-II-dependent antigen-presenting activity of macrophages, with a focus on the orchestration of effector immune responses [59]. In addition, gal-1 has been shown to contribute to the anti-inflammatory state during the resolution of inflammation by promoting the generation of M2-like macrophages both in vivo and ex vivo [60] (Fig. 2). However, the broader implications of gal-1’s glycosylation-modifying activity, including regulating other glycoproteins implicated in macrophage polarization, remain underexplored. Addressing these questions could illuminate novel pathways for modulating macrophage function in pathological pregnancies, particularly those characterized by excessive inflammation.

Another important leukocyte population at the feto-maternal interface is DCs. In mice, the number of uterine DCs increases in early pregnancy, and the highest number is reached during mid-pregnancy and plateaus until term [61]. Decidual DCs acquire a tolerogenic phenotype to promote immune tolerance at the feto-maternal interface. Exposure to gal-1 induces regulatory signatures in DCs, including the expression of CD45RB and secretion of interleukin (IL)-10 and IL-27, which are crucial for maintaining immune tolerance [46, 62]. In line with this finding, we have shown that the absence of gal-1 during pregnancy skews DCs toward an inflammatory phenotype and impairs immune tolerance [27]. In human DCs, microarray analysis revealed that gal-1 influenced a subset of genes relevant to cell migration [63]. Moreover, in vitro experiments showed that recombinant gal-1 promoted the migration and maturation of murine bone marrow-derived DCs, as indicated by the upregulation of their cell surface maturation markers CD80 and CD86 and by their IL-6 production [64]. Collectively, these findings demonstrate that gal-1 promotes maternal immune tolerance by modulating dendritic cell function and limiting inflammatory responses at the feto-maternal interface.

In early human pregnancy, decidual T cells constitute 5–20% of total CD45^+^ decidual lymphocytes, and this percentage increases with gestational age to 40–80% [65]. It was reported that gal-1 induced the apoptosis of decidual but not peripheral alloreactive T cells, presenting a distinct glycophenotype compatible with sensitivity to gal-1 [28]. Importantly, gal-1 selectively induces apoptosis in T helper (Th)1 and Th17 cells, while sparing Th2 and Treg cells. This selective susceptibility is attributed to distinct glycosylation patterns on the surface of Th1 and Th17 cells. Specifically, the high abundance of terminal α2,3-sialylated glycan ligands on these subsets facilitates gal-1 binding, rendering them more prone to gal-1-mediated apoptosis [66]. Furthermore, an indirect effect of gal-1 on Tregs was presented in a thorough study in which the treatment of *Lgals1*^*−/−*^ mice with recombinant gal-1 prevented fetal loss and restored tolerance through the induction of tolerogenic DCs, which subsequently promoted the expansion of IL-10‒secreting Tregs [27]. Overall, the selective apoptosis of Th1/Th17 cells and IL-10-secreting Treg expansion is likely mediated by differential glycosylation patterns on T cell subsets, underscoring gal-1’s strategic role in tipping the balance toward immune tolerance.

Placental galectins, such as gal-13, gal-14, and gal-16, are uniquely expressed in the placenta and play important roles in immune modulation. While gal-1 is well characterized for its pleiotropic immune functions in vitro and in vivo [27, 67], the limited availability of animal models has constrained in vivo research on placental galectins. When added exogenously, gal-13 and gal-14 induce apoptosis in activated T cells, like gal-1 [10], with a stronger effect on cytotoxic T (Tc) cells compared to Th cells [68], likely due to differential glycosylation [69]. Placental galectins also increased cell surface CD95 expression of T lymphocytes, similar to gal-1 [70], potentially heightening sensitivity to activation-induced cell death, while modulating proliferation markers, such as CD25 and CD71 [68]. Gal-16 was also recently found to induce the apoptosis of activated T cells [10]. Recombinant gal-16 has been found to bind strongly to the c-Rel which might prevent c-Rel to induce gene expression of anti-apoptotic genes, thus promoting T-cell apoptosis during pregnancy [71]. In contrast to earlier reports where pre-activated cells and long-term treatment were used, short-term treatments of non-activated peripheral blood mononuclear cells (PBMCs)—including monocytes, T cells, B cells, as well as NK cells—with gal-13 and gal-14 result in reduced apoptosis [72]. This indicates that placental galectin-induced apoptosis is strongly influenced by the activation status of target cells. Placental galectins also enhance PBMCs’ cytokine production, such as pro-angiogenic IL-8, tolerogenic IL-10, and pro-inflammatory interferon (IFN)-γ which latter inhibits Th17 differentiation as well as promotes decidual natural killer (dNK) cell differentiation and placental development [73–75]. Furthermore, gal-13 and gal-14 activate key signaling pathways involved in immune regulation and cell longevity, such as Erk1/2, p38 mitogen-activated protein kinase (MAPK), and nuclear factor kappa-B (NF-ĸB) [59, 76–80].

Interestingly, gal-13 does not induce apoptosis in neutrophils but shifts them towards a placental-growth-permissive phenotype, maintaining their primary immune functions while supporting placental growth [81]. These alterations include induced reactive oxygen species (ROS) production and the expression of CD66b, CD11b, programmed death-ligand 1 (PD-L1), hepatocyte growth factor (HGF), vascular endothelial growth factor (VEGF), matrix metalloproteinase 9 (MMP-9), and tumor necrosis factor-α (TNF-α). This phenomenon has been observed with increased migration of neutrophils into the decidua during early pregnancy [81, 82], further highlighting the complex immune modulation at the feto-maternal interface. Together, these findings suggest that, depending on their differentiation and activation status, galectin-1 and placental galectins exert both overlapping and distinct effects on various immune cell types, thereby contributing to the fine-tuning of the immune environment at the feto-maternal interface.

### Galectin-1 and placental galectins from embryo implantation to trophoblast differentiation and placental development

Although galectins’ immunoregulatory roles during gestation are well studied, their involvement in placental development, angiogenesis, and trophoblast function is more recent. Maternal gal-1 promotes endometrial receptivity [83], and reduced *LGALS1* expression is linked to unexplained infertility [84]. On the other side, fetal gal-1, present in the blastocyst trophectoderm and inner cell mass, may assist embryo attachment [23]. In mice, gal-1 binds fibroblast growth factor receptors (FGFR1-4) [85], crucial for trophoblast lineage decision, corroborated by failed differentiation of *Fgfr1*^*−/−*^ trophectoderm cells and nonviability of *Fgfr1*^*−/−*^ or *Fgfr2*^*−/−*^ blastocysts [86]. Furthermore, trophoblast stem cells (TSCs) upregulate gal-1 when cocultured with endometrial cells, and exogenous gal-1 promotes their fusion, migration, and invasion [87] (Fig. 2). All these results highlight the importance of gal-1 in embryo implantation; however, some findings warrant further investigation in pregnancy-related model systems. A more systematic analysis of embryo implantation rates and gal-1 expression in the context of IVF could provide deeper insights into its role in facilitating implantation and early embryo–endometrium communication.

Placental development is governed by trophoblast lineage differentiation, a process in which galectins play an active role. For example, gal-1 promotes the fusion of villous CTBs into STB by inhibiting β-catenin, E-cadherin, and Ki67 [88]. Silencing *LGALS1* has been shown to impair syncytium formation [89]. Additionally, gal-1 is detected at the STB brush border, suggesting a role in modulating maternal immune responses, although at much lower levels than maternal gal-1 [26].

Placental galectins (gal-13, gal-14, and gal-16) are upregulated during CTB differentiation, driven by key TFs involved in trophoblast fusion [39], activated via cyclic AMP, protein kinase A, and p38 mitogen-activated kinase pathways [90, 91]. These TFs are downregulated in preterm severe preeclampsia (PE) with intrauterine growth restriction (IUGR), potentially reducing placental galectin expression and impairing placentation [39]. It remains unclear whether the downregulation of galectins in such cases has an autocrine inhibitory effect on placentation. Additionally, *LGALS16* mRNA appears by day 7–8 in the polar trophectoderm [92], responsible for implantation and CTB differentiation [93]. These data collectively suggest that gal-1 and placental galectins are important players in implantation and placenta development (Fig. 2). However, further studies are needed to elucidate both the individual and synergistic roles of gal-1 and placental galectins within the molecular and cellular networks that regulate trophoblast differentiation, as current research often focuses on individual galectins while overlooking the simultaneous expression and interaction of other family members.

In the trophoblast lineage, galectins play a crucial role in regulating the invasive differentiation pathway of EVTs [94, 95]. RNA-seq confirmed abundant *LGALS1* expression in EVTs, which contributes to spiral artery remodeling via interactions with decidual cells [22, 96]. Gal-1, highly expressed in EVT cell columns, binds extracellular matrix components like laminin and fibronectin, influencing ECM organization and cell adhesion in the placental bed [97, 98]. The expression pattern of gal-1 in the first-trimester placenta and the blockade of endogenous gal-1, which substantially abrogates the migration of trophoblasts and in vitro [3, 95, 99], both propose that gal-1 modulates the EVT differentiation and invasion. Gal-1 also modulates HLA-G expression, affecting both soluble and membrane-bound forms, contributing to a self-regulatory mechanism for EVT invasion as they approach maternal vasculature, given soluble HLA-G’s inhibitory role on invasion [23, 100].

Among placental galectins, gal-14 promotes trophoblast migration and invasion by upregulating MMP-9 and N-cadherin in EVTs via Akt phosphorylation [101]. It was speculated that this process is NF-kB-dependent because gal-14 significantly colocalizes with c-Rel, a TF that is enriched in EVTs and possesses binding sites in the *MMP9* promoters [80, 102, 103]. In addition, overexpressed gal-13 binds homeobox A1 (HOXA1), another EVT-related TF [37, 104, 105]. However, these two latter findings are based on overexpression in non-placental cells, and the naturally low expression of placental galectins in EVTs calls for further validation in EVT models. Furthermore, beyond autocrine regulation, paracrine effects have been proposed for gal-13. Crystal-like gal-13 (PP13) aggregates in the decidua and necrotic immune cells in their vicinity have been observed in first-trimester placental histological Sect. [106]. This may indirectly promote EVT invasion by redirecting maternal immune cells away from spiral arterioles (Fig. 2), though this too requires functional confirmation.

## Placental evolutionary advances and ligands – prototype galectins

Placentation is a defining trait of eutherian mammals, enabling nutrient and signal exchange between mother and fetus. In humans and mice, the hemochorial placenta, where trophoblasts directly contact maternal immune cells, is the most invasive type. While structurally different across species [107], this invasiveness exposes fetal cells to maternal immune responses, making tight immune regulation essential for tolerance, proper placental access to blood, and protection against excessive invasion or infection [108–110]. Species with invasive placentation, like humans and mice, show stronger maternal immune regulation than those with non-invasive types, such as epitheliochorial placentation [111], suggesting these mechanisms evolved early through adaptation of existing immunoregulatory pathways.

**Fig. 4:**
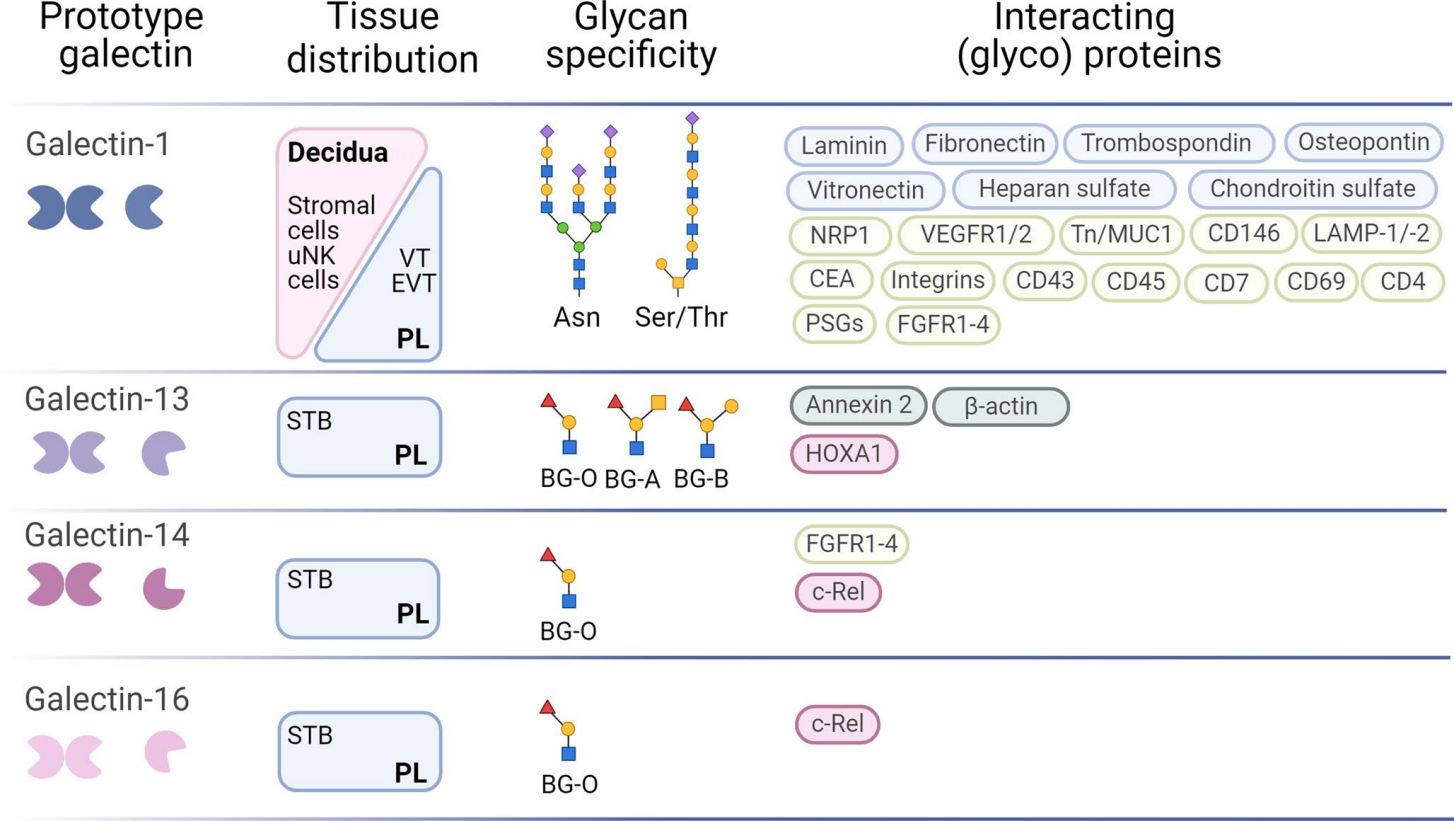
Main sources, specificity, and interacting ligands of gal-1, gal-13, gal-14, and gal-16 at the feto-maternal interface. Summary of tissue distribution, glycan specificity, and carbohydrate-dependent and carbohydrate-independent interaction partners (blue: extracellular matrix proteins, light green: plasma membrane glycoproteins, dark green: intracellular proteins, pink: transcription factors) of gal-1 and placental galectins in mammalian pregnancies. BG, blood group; CRD, carbohydrate recognition domain; EVT, extravillous trophoblast; PL, placenta; SC, stromal cell; STB, syncytiotrophoblast; uNK, uterine natural killer; VT, villous cytotrophoblast. Mannose  Galactose  N-acetylglucosamine  N-acetylgalactosamine  Fucose  Sialic acid

Galectin ligands play an important role in modulating the maternal-placental dialogue to regulate the immune system, contributing to a successful pregnancy outcome. Although several ligands have been described, we focus on pregnancy specific glycoprotein-1 (PSG1) in the current review. PSG1 is a highly glycosylated STB- and EVT-derived, pregnancy-specific protein in the maternal circulation [112–115] (Fig. 4). Reduced PSG1 expression has been associated with pregnancy complications such as IUGR and PE [30, 112, 116]. Recent study by Mendoza et al. demonstrated that gal-1 binds to the N- and A2-domains of PSG1 in a glycan-dependent manner, which protects gal-1 from oxidative inactivation [115]. This binding is facilitated by N-glycosylation, particularly complex-type N-glycans with α2,3-linked sialic acid, which enhances gal-1 binding [115]. The *PSG1* gene is a member of a large gene family on human Chr19, which also includes genes involved in feto-maternal immune regulation and placentation. This gene cluster likely evolved in species with invasive hemochorial placentation, providing immunoregulatory and proangiogenic functions that support successful placentation [117]. Importantly, Chr19 also hosts other large gene clusters involved in the regulation of feto-maternal immune interactions and placentation, such as those encoding human chorionic gonadotropins (*CGBs)*, Chr19 microRNAs, and placental galectins [10].

The emergence of the diverse galectin family on Chr19 in anthropoid primates was driven by gene duplication and deletion events, also called birth and death evolution, facilitated by repetitive elements [10, 118, 119]. Furthermore, three of Chr19 galectins (gal-13, gal-14, and gal-16) which are placenta-specific and play crucial roles in placental function, have acquired placenta-specific regulatory elements in their promoters during primate evolution [39]. While placental galectins share a common galectin fold, their CRDs have evolved distinct carbohydrate-binding specificities, showing a preference for β-galactosides, particularly LacNAc and GalNAc, which are present on STB apical membranes, as well as blood carbohydrate antigens [3, 10, 11, 38, 39, 120, 121] (Fig. 3). Although few studies suggested a low affinity for lactose, recent findings indicate that gal-13 and other galectins bind to asialofetuin, a glycoprotein containing complex carbohydrates [68, 122]. Discrepancies in these findings may be attributed to methodological differences or variations in the types of carbohydrates studied. This highlights the need for further research to clarify the exact carbohydrate-binding properties of placental galectins.

The rapid neofunctionalization of placental galectins may have been driven by an evolutionary arms race with pathogens, as maintaining immune tolerance while defending against infections is critical in the unique feto-maternal environment [123, 124]. Placenta-specific galectins, predominantly expressed by the STB, are positioned at the interface where fetal tissues interact with maternal immune cells and potential pathogens in the maternal circulation. As discussed above, multiple studies have provided evidence for the immunoregulatory effects of these galectins on maternal immune cells and for the question of whether these galectins can also interact with invading pathogens, similar to other galectins (e.g., the interaction of gal-1 with *Haemophilus influenzae* and HIV-1) [125–127]. Additionally, placental galectins are present in EVs secreted by the STB, which may facilitate short-distance communication between the placenta and the mother, offering another layer of regulatory functions [128–130]. The evolution of placental galectins in anthropoid primates with invasive placentation likely represents an adaptation that supports immune tolerance at the feto-maternal interface, enabling prolonged gestation and successful pregnancy despite the challenges posed by fetal semi-allografts [10].

## Dysregulation of galectin-1 and placental galectins in pregnancy pathologies

**Fig. 5:**
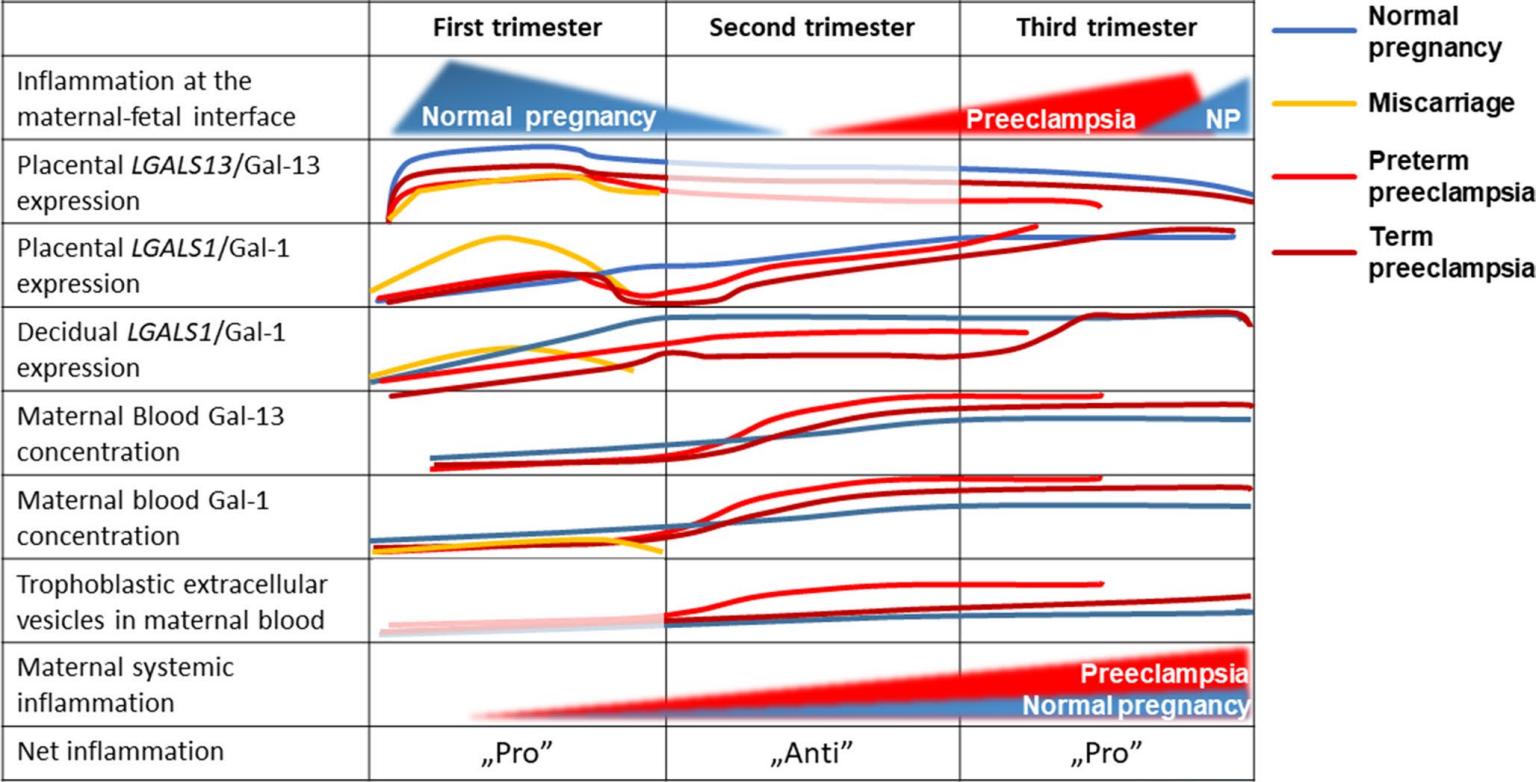
Altered expression and maternal blood levels of gal-1 and gal-13 in adverse pregnancy outcomes. The figure consolidates the results of numerous studies on various facets of gal-1 and gal-13 in normal pregnancies, preeclampsia, and miscarriage. The placental expression of *LGALS13* and gal-13 is strong in the first trimester, and the secreted protein can be detected in maternal blood beginning at the 5th week of gestation in healthy pregnancies. Gal-1 is highly expressed in the decidua and less abundant in the placenta, and its maternal blood concentration increases in the first and second trimesters. In the second and third trimesters, the maternal serum gal-13 concentration increases due to increasing placental volume, paralleling the increase in maternal systemic inflammation. In preeclampsia, especially in early-onset cases, there are lower placental expressions and maternal serum concentrations of gal-1 and gal-13 in the first trimester, coincident with impaired trophoblast invasion and spiral artery remodeling. Starting in the second trimester, ischemic placental stress and proinflammatory changes at the feto-maternal interface are also reflected by increased shedding of aponecrotic microvesicles, which carry a considerable amount of gal-13, elevating maternal blood gal-13 concentrations. In miscarriages, placental gal-1 and gal-13 expression are lower, and there is less circulating gal-1 in maternal blood. Figure adapted from [38], © under the terms Creative Commons Attribution License (CC BY)

The expression of immunoregulatory molecules (including galectins) at the feto-maternal interface is crucial for maintaining proper immune regulation during pregnancy. Dysregulation of maternal and placental galectins has been linked to various pregnancy complications, collectively known as the “great obstetrical syndromes”, including miscarriage, preterm birth, PE, IUGR, gestational diabetes, and ectopic pregnancy [43, 131–134]. These complications often involve disturbances in immune tolerance and proinflammatory processes, with the altered expression of galectins (e.g., gal-1, gal-13, gal-14, and gal-16) playing pivotal roles. This section focuses on the dysregulation of these galectins in miscarriage, PE, and IUGR, particularly those associated with defective placentation and impaired feto-maternal immune tolerance (Fig. 5).

### Miscarriages

Miscarriage, a significant reproductive challenge, occurs in 50–80% of women who conceive, with clinically recognized pregnancies affected in 15–25% of cases [135, 136]. Studies in animal models, particularly those with mutations in the P4 nuclear receptor, have demonstrated that P4 facilitates the transcription of genes involved in uterine receptivity, implantation, decidualization, and pregnancy maintenance. Notably, the expression of galectin (*LGALS1/Lgals1*, *LGALS13*, and *LGALS14*) genes is regulated by P4 [26, 39] and estrogen [26, 39, 137], suggesting a complex interaction between these ovarian steroids and galectin expression during pregnancy. Accordingly, altered expression of galectins (gal-1, gal-13, and gal-14), has been observed in miscarriages both at the mRNA and protein levels where P4 and estrogen levels or the expression of their receptors are also found decreased [138, 139]. For example, we have reported lower placental expression of gal-13 and gal-14 in miscarriage cases [68], a finding consistent with others who have noted a decrease in gal-1 levels in the villous placenta and maternal circulation in these patients [23, 140, 141]. Moreover, studies indicate a significant interplay between P4, PIBF, and gal-1 at the feto-maternal interface, promoting immune tolerance and pregnancy maintenance [27]. Based on these evidences, it has become clear that ovarian steroid deficiency leads to galectin deficiencies in the decidua and/or placenta, resulting in impaired immune tolerance and possible autoimmune or alloimmune rejection, contributing to pregnancy loss [142, 143].

### Preeclampsia and intrauterine growth restriction

Preeclampsia, affecting approximately 3–8% of pregnancies, is a heterogenous syndrome characterized by systemic inflammation, endothelial dysfunction, and anti-angiogenesis state, with early-onset forms linked to severe outcomes [144]. Recent molecular profiling has revealed four PE subclasses including a proinflammatory “maternal anti-fetal rejection-type” subclass, marked by proinflammatory components [144–146]. This subclass highlights the role of galectins, especially those with anti-inflammatory and proangiogenic properties, in the pathogenesis of PE. In particular, gal-1 has been shown to be upregulated in EVT and the decidua during PE, reflecting a possible fetal response to maternal systemic inflammation [132, 147]. We and others have also shown that soluble gal-1 levels in maternal circulation are decreased in the first and second trimester in women who later develop PE, with levels rising in the third trimester as clinical symptoms manifest [148, 149]. Current results support these findings regarding soluble gal-1; however, changes to gal-1 were not observed in the EV fraction of maternal plasma [130]. Thus, the dynamic expression pattern is consistent with the role of gal-1 serving as a marker of both the progression and severity of PE. In addition, experimental models, including *Lgals1* knockout mice, have demonstrated that loss of gal-1 expression results in PE-like features, such as exacerbated inflammation, endothelial dysfunction, and restricted placental and fetal growth [148]. These findings demonstrate that impaired maternal gal-1 expression at the feto-maternal interface, especially early in pregnancy, may disrupt immune tolerance and contribute to the development of PE.

Additionally, placental development failure in early-onset/preterm PE has been well documented [150, 151]. Reduced expression of *LGALS13* (gal-13) in the first trimester has been associated with the later development of PE, with lower gal-13 concentrations found in the maternal blood and placenta [152–154]. Recent meta-analyses have highlighted gal-13 (PP13) as a potential biomarker for early screening of PE, showing its predictive power when used in combination with other biomarkers [38, 155]. In preterm PE, decreased placental expression of gal-13 and gal-14 in the third trimester has been noted [39, 129, 156, 157], while soluble gal-13 and gal-13-containing EVs are elevated in maternal circulation [128, 129, 158]. This suggests that placental stress may lead to the increased shedding of these galectins into the maternal bloodstream, potentially exacerbating immune dysfunction [129, 156]. This latter study also showed that the quantity of EV-associated gal-13 is greater than that of soluble gal-13 [128], suggesting that the vesicular secretion of this and potentially other placental galectins might be the major route for their liberation into the maternal circulation. Of note, it has been found that the actin cytoskeleton is involved in the regulation of trophoblastic unconventional gal-13 export [120], most likely in conjunction with lipid rafts [156].

Furthermore, genetic variations in the *LGALS13* gene, such as the − 98 A/A promoter single-nucleotide polymorphism (SNP), have been linked to reduced gal-13 expression and an increased risk of PE [39, 159]. Functional deficiency of gal-13 due to polymorphisms, such as the Dex-2 and delT221 variants, further supports the hypothesis that reduced galectin function at the feto-maternal interface predisposes individuals to severe forms of PE, particularly early-onset cases [38, 160]. Such research is limited for placental galectins and has not yet been performed for gal-1, therefore, further studies are warranted to explore the association of *LGALS1*, *LGALS13*, *LGALS14*, and *LGALS16* polymorphism with miscarriage, PE, or IUGR.

IUGR, affecting 5–10% of all pregnancies worldwide [161], can occur independently or be associated with PE. Studies on gal-1 in IUGR yielded conflicting results. One study found no difference in gal-1 staining of the placenta between patients and controls [162], whereas another report showed lower placental and maternal serum gal-1 levels in IUGR without comorbidities compared to healthy controls [163]. Regarding placental galectins, gal-13 expression was reduced in IUGR placentas compared to controls, only in the case of women carrying male but not female fetuses [162]. Additionally, first-trimester gal-13 levels in maternal serum were lower in women who subsequently developed IUGR compared to gestational age-matched controls [154]. However, future research is warranted to explore the potential role of galectins in IUGR pathogenesis.

## Future research and challenges

Gal-1 and placental galectins are established as key players in pregnancy biology and immunology, offering valuable potential for the diagnosis and therapeutic management of pregnancy-related complications. However, several challenges remain in fully understanding their roles and translating this knowledge into clinical applications. Future research should address the following key areas:

1. *Pathophysiological mechanisms*: A deeper understanding of the precise roles of maternal and placental galectins in the pathophysiology of pregnancy complications is critical. Future studies should explore how maternal and placental galectins contribute to the development of conditions such as miscarriage, PE, and IUGR. Investigating their involvement in immune regulation, placental development, and feto-maternal interactions could reveal new insights into the mechanisms that underlie these disorders.
2. *Functional characterization*: Further in vitro and in vivo research is necessary to characterize the functional effects of maternal and placental galectins in relevant cell types. These studies should aim to elucidate their pleiotropic roles in pregnancy, including potential synergistic or antagonistic effects, especially through heterodimerization with other galectins, the so-called galectin signature. Understanding the diverse functions of maternal and placental galectins, both individually and in combination, will be critical for uncovering their full spectrum of activities in pregnancy.
3. *Standardization of methodologies*: A major challenge lies in the need for standardized patient sampling and experimental methodologies. To establish the true diagnostic and prognostic value of maternal and placental galectins in adverse pregnancy outcomes, uniform protocols are essential for accurate measurement and comparison across studies. Furthermore, simultaneous measurement of total, free, and EV-bound galectin levels from the maternal blood in a standardized fashion could provide valuable insights into the health state of the feto-maternal interface. This would allow for reliable biomarker identification and consistent results across different patient populations.
4. *Biomarkers for adverse pregnancy outcomes*: Future research should investigate whether altered levels or patterns of galectin expression, either in soluble form or associated with EVs, can serve as early indicators of pregnancy complications. Identifying specific galectin profiles linked to pathological conditions may aid in early diagnosis, risk stratification, and improved monitoring of maternal–fetal health.

## Conclusions

In summary, galectins are essential for regulating feto-maternal interactions during pregnancy, supporting implantation, placental development, deep invasion into maternal tissues, as well as immune tolerance towards the semi-allogeneic fetus and placenta. Maternal gal-1 emerged early in placental mammalian evolution, while placental galectins evolved more recently in primates to enhance immune tolerance and deep placentation, aiding fetal nutrition and brain development. Disrupted galectin expression and function are linked to severe obstetrical syndromes, highlighting their potential for future diagnostic and therapeutic applications.

## Data Availability

The generated dataset is available from the corresponding author on reasonable request.
